# Impact of Enhanced Recovery After Surgery on Postoperative Recovery for Pancreaticoduodenectomy: Pooled Analysis of Observational Study

**DOI:** 10.3389/fonc.2019.00687

**Published:** 2019-07-30

**Authors:** Yang Cao, Hui-Yun Gu, Zhen-Dong Huang, Ya-Peng Wu, Qiong Zhang, Jie Luo, Chao Zhang, Yan Fu

**Affiliations:** ^1^Center for Evidence-Based Medicine and Clinical Research, Taihe Hospital, Hubei University of Medicine, Shiyan, China; ^2^Department of Surgery, Zhongnan Hospital of Wuhan University, Wuhan University, Wuhan, China; ^3^Department of Surgery, Taihe Hospital, Hubei University of Medicine, Shiyan, China

**Keywords:** pancreaticoduodenectomy, enhanced recovery after surgery, mortality, postoperative complications, delayed gastric emptying

## Abstract

**Purpose:** To assess the impact of enhanced recovery after surgery (ERAS) protocols in pancreaticoduodenectomy.

**Methods:** Four databases were searched for studies describing ERAS program in patients undergoing pancreatic surgery published up to May 01, 2018. Primary outcomes were mortality, readmission, reoperation and postoperative complications. Secondary outcomes were the length of stay and cost.

**Results:** A total of 19 studies met inclusion and exclusion criteria and included 3,387 patients. Meta-analysis showed a decrease in pancreatic fistula (OR = 0.79, 95% CI: 0.67 to 0.95; *I*^2^ = 0%), infection (OR = 0.63, 95% CI: 0.50 to 0.78; *I*^2^ = 0%), especially incision infection (OR = 0.62, 95% CI: 0.42 to 0.91; *I*^2^ = 0%), and pulmonary infection (OR = 0.28, 95% CI: 0.12 to 0.66; *I*^2^ = 0%). Length-of-stay (MD: −3.89 days, 95% CI: −4.98 to −2.81; *I*^2^ = 78%) and cost were also significantly reduced. There was no significant increase in mortality, readmission, reoperation, or delayed gastric emptying.

**Conclusion:** This analysis revealed that using ERAS protocols in pancreatic resections may help decrease the incidence of pancreatic fistula and infections. Furthermore, ERAS also reduces length of stay and cost of care. This study provides evidence for the benefit of ERAS protocols.

## Introduction

The concept of enhanced recovery after surgery (ERAS) (1–3) was firstly applied in colorectal surgery and is increasingly applied to other surgical fields, such as gastric (4) and orthopedic (5) surgeries. In 2013, guidelines for perioperative care for pancreaticoduodenectomy (PD) were published by the European Society for Clinical Nutrition and Metabolism and the International Association for Surgical Metabolism and Nutrition; these guidelines contain 27 care items and change to three aspects; preoperation, intraoperation, and postoperation (6). The purpose of these changes was to reduce patients' stress responses and time-to-recovery by close cooperation between surgeons, anesthesiologists, intensive care workers and nurses (7).

At present, pancreaticoduodenectomy is one of the major treatments for malignancies such as pancreatic cancer, periampullary cancer and endocrine neoplasm (8). PD is a technically complex and subtle operation, which has been performed with increasing frequency and decreased mortality rates (9) using ERAS protocols over the past few years. However, morbidity rates have remained high (30–60%) (10). Four meta-analyses confirmed that ERAS can reduce length-of-stay (LOS) and hospital costs; one meta-analysis published in 2013 (11) indicated that the incidence of delayed gastric emptying (DGE) and pancreatic fistula (PF) did not differ significantly between groups, whereas the other three, published in 2015 (12), 2016 (13), and 2018 (14) found that the incidence of DGE was lower in the ERAS groups. In a study from 2015, additional outcome measures were used, and postoperative complication rate and mortality, were reduced in the ERAS groups. Another article published in 2018 (14) mentioned that ERAS has a lower incidence of the mild complications, and abdominal infection. Therefore, ERAS programs in patients undergoing PD have not been completely analyzed, and the use of various outcome measures in different studies increases the difficulty of comparison.

To solve this problem, we need to clarify the real impact of ERAS protocols in this study. The purpose of this meta-analysis was to evaluate the influence of ERAS programs for patients undergoing PD and to provide information for establishing reliable predictions for clinical treatment outcomes.

## Methods

### Selection of Studies

Our search used the guidelines of Preferred Reporting Items for Meta-analysis (15). We obtained a list of eligible studies from the following databases: Ovid MEDLINE, OVID EMBase, the Cochrane Library, and ISIWeb of Science, published in English up to May 01, 2018. The search strategy is shown in Supplemental Method 1.

### Inclusion and Exclusion Criteria

Studies were included in the meta-analysis if the following criteria were met: studies that involved patients undergoing PD, pylorus-preserving pancreatoduodenectomy (PPPD), pancreaticojejunostomy, proximal pancreatic resection, or distal pancreatectomy, approached either with open or minimally invasive surgery; studies that included both an ERAS group and a conventional group, treated by ERAS protocols and conventional care, respectively; studies that reported outcomes such as mortality (in-hospital death, irrespective of duration of stay, or death occurring within 30 days of discharge), reoperation and hospital readmission, various types of fistula such as pancreatic fistula (16) [PF, according to the International Study Group on Pancreatic Fistula (ISGPF), defined as any measurable amount of drainage fluid, with amylase three times the normal level, on or after postoperative day 3], anastomosis leakage, biliary fistula, chylous fistula, intestinal fistula, different types of infections, DGE (17) (need for nasogastric decompression or vomiting occurring), length of hospital stay (LOS) including the postoperative LOS and total LOS and/or costs. Primary outcome measures were mortality, reoperation, readmission, and postoperative complications; complications mainly cover fistula, infection, and DGE. Other outcomes were seen as secondary outcome. The type of study design was observational study.

Studies meeting any of the following selection criteria were excluded: (1) the language is not English, (2) repetitive studies, (3) unobtainable source literature or original data cannot be obtained from the literature, (4) emergency operations, and (5) total pancreatectomy.

### Data Extraction and Quality Assessment

Relative data were extracted by two independent authors (Cao and Huang) with a unified standard. Differences or contradictions between the authors were resolved by discussion or consultation of a third investigator (Gu). The extracted variables include country of author; publication year; study design; the age and gender of patients; follow-up time; operation; LOS; mortality; readmission and complications, including fistula, infection, and DGE. Hospital costs were also extracted from the articles, if possible. Methodological quality of the studies was assessed using the Newcastle-Ottawa Scale (NOS) (18, 19) with eight items. A study can be rewarded a maximum of nine stars, with a maximum of two stars for Comparability and one star for each numbered item within the Selection and Exposure categories. More than six stars indicate a study of high quality.

### Assessment of Bias

Identified studies were roughly divided into 2 types, either cohort studies or case-control studies, and were assessed using the NOS with the accompanying coding manual for bias. Two authors (Cao and Huang) were independently responsible for assessment of bias.

### Statistical Analysis

Meta-analysis was conducted by using the R Programming Language. Dichotomous variables mainly used odds ratio (OR) for mortality, reoperation, readmission, various fistula, infection, and DGE and 95% confidence intervals (CI) obtained by standard technique (20). Mean difference (MD) and standard deviation were calculated for continuous variables. The results were presented graphically using forest plots. Heterogeneity (21) of the included results was detected by *I*^2^. If *I*^2^ ≥ 40%, we chose the random effect model, else we selected the fixed effect model. The *I*^2^ statistics represents the amount of variability in the meta-analysis attributed to study heterogeneity. All analyses were conducted with a significance level of 0.05 (22). To determine the source of heterogeneity, results of fistula, infection, DGE, and LOS were analyzed by subgroup; fistula and infection were classified according to type, DGE was divided according to severity, and LOS was divided into preoperative and total time, which can determine the source of heterogeneity.

## Results

### Literature Identification

**Figure 1:**
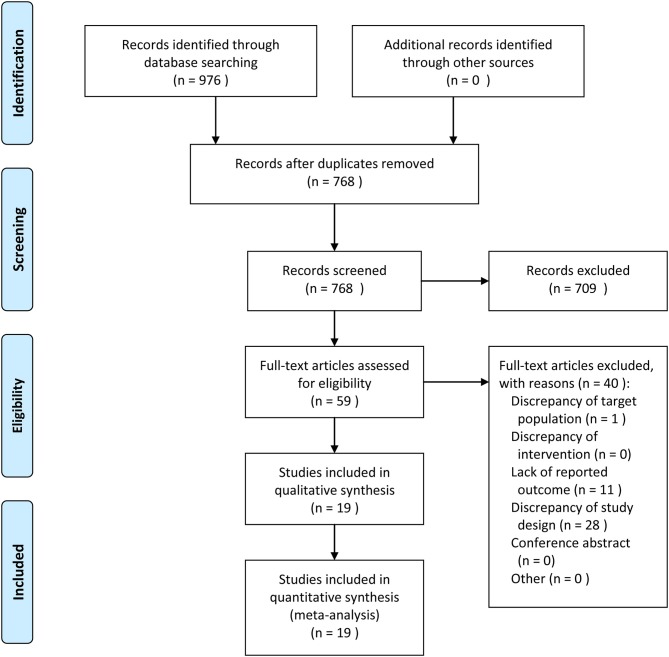
Meta-Analyses (PRISMA) flow diagram depicting the process of identification and inclusion of selected studies.

The flow of study identification and inclusion is shown in Figure 1. The initial search resulted in 976 abstracts. After removing 208 duplicate studies, 768 potentially relevant studies were selected on the basis of the abstract. Then, 709 studies were further excluded on the basis of the abstract, and the full texts of the remaining 59 articles were assessed for eligibility. An additional 40 articles (Supplemental Table 1) were excluded. Finally, 19 articles were included in this study.

### Study Characteristics

The characteristics of the 19 included articles, which comprised 7 cohort studies (8, 23–28) and 12 case-control studies (7, 29–39), are shown in Table 1, which totally contains 3387 patients. Thirteen studies (8, 23, 26, 28–34, 37–39) included patients undergoing PD, one study (35) included patients undergoing distal pancreatectomy (DP), one study (25) included patients undergoing proximal pancreatic resection, one study (36) included patients undergoing laparoscopic pancreatoduodenectomy (LDP), and three studies (7, 24, 27) included patients undergoing two forms of pancreatectomy.

**Table 1:** Study characteristics.

**References**	**Study design**	**Group**	**Age (years)**	**Male/female**	**Operations**	**Follow-up time (months)**	**Sample size**	**Country**
Balzano et al. (23)	Cohort study	ERAS	64.3 ± 13.75	155/97	PD	36	252	England
		CPC	62.9 ± 14.5	148/104	PD	48	252	
French et al. (25)	Cohort study	ERAS	53.8 ± 11.6	NA	PPR	18	9	England
		CPC	66.2 ± 10.3	NA	PPR	18	49	
Abu Hilal et al. (29)	Case-control	ERAS	68.5 ± 5.58	10/10	PD	15	20	England
		CPC	68.92 ± 11.97	10/14	PD	15	24	
Nikfarjam et al. (33)	Case-control	ERAS	65.5 ± 9	13/7	PD	88	20	Australia
		CPC	55 ± 16.5	12/9	PD	88	21	
Braga et al. (31)	Case-control	ERAS	69 ± 2.17	66/49	PD	26	115	Italy
		CPC	69 ± 2.17	66/49	PD	33	115	
Coolsen et al. (7)	Case-control	ERAS	67 ± 11	44/42	PD/PPPD	24	86	Netherlands
		CPC	62 ± 13	58/39	PD/PPPD	120	97	
Kobayashi et al. (32)	Case-control	ERAS	67.5 ± 10.7	61/39	PD	36	100	Japan
		CPC	65.4 ± 10.8	62/28	PD	48	90	
Pillai et al. (8)	Cohort study	ERAS	44.2 ± 15.9	9/11	PD	8	20	India
		CPC	47.6 ± 12.0	10/10	PD	NA	20	
Williamsson et al. (38)	Case-control	ERAS	69 ± 16.25	31/19	PD	NA	50	Sweden
		CPC	67 ± 14	26/24	PD	36	50	
Richardson et al. (36)	Case-control	ERAS	63.41 ± 12.68	9/13	LDP	19	22	England
		CPC	56.81 ± 22.22	20/24	LDP	48	44	
Shao et al. (27)	Cohort study	ERAS	56.96 ± 11.50	194/131	PD/PPPD	24	325	China
		CPC	57.05 ± 12.30	184/126	PD/PPPD	24	310	
Zouros et al. (39)	Case-control	ERAS	65.9 ± 10.5	46/29	PD	48	75	Greece
		CPC	63.9 ± 11.6	34/16	PD	48	50	
Shah et al. (37)	Case-control	ERAS	61.9 ± 9.1	84/58	PD	50	142	India
		CPC	59.1 ± 10.4	30/16	PD	28	46	
Partelli et al. (34)	Case-control	ERAS	77.75 ± 1.75	14/8	PD	NA	22	Italy
		CPC	78 ± 1.75	33/33	PD	NA	66	
Bai et al. (30)	Case-control	ERAS	58 ± 13	69/55	PD	15	124	China
		CPC	57 ± 12	37/26	PD	9	63	
Dai et al. (24)	Cohort study	ERAS	58.5 ± 12.75	34/34	PD/PPPD	28	68	China
		CPC	58.2 ± 11.5	51/47	PD/PPPD	28	98	
van der Kolk et al. (28)	Cohort study	ERAS	64.59 ± 12.04	56/39	PD	24	95	Netherlands
		CPC	65.29 ± 10.67	35/13	PD	36	52	
Pecorelli et al. (35)	Case-control	ERAS	62.4 ± 13.4	49/51	DP	48	100	Italy
		CPC	60.4 ± 13.8	44/56	DP	48	100	
Kagedan et al. (26)	Cohort study	ERAS	65 ± 13.51	74/47	PD	12	121	Canada
		CPC	65.85 ± 12.10	31/43	PD	18	74	

*ERAS, Enhanced Recovery after Surgery; CPC, conventional perioperative care; PD, Pancreaticoduodenectomy; PPR, proximal pancreatic resection; PPPD, pylorus-preserving pancreatoduodenectomy; DP, Distal pancreatoduodenectomy. Values of Age are mean ± SD*.

### ERAS Characteristics

Characteristics of these studies are shown in Table 2. The most common ERAS interventions in the studies were preoperative counseling, antimicrobial prophylaxis and skin preparation, epidural analgesia, postoperative artificial nutrition, and early and scheduled mobilization. That was followed by anti-thrombotic prophylaxis, postoperative nausea and vomiting (PONV) and avoiding hypothermia. However, none of the studies reported on perioperative biliary drainage, preoperative smoking, wound catheters or transversus abdominis plane block, alcohol consumption, or somatostatin analogs.

**Table 2:** ERAS characteristics.

**References**	**Group**	**Enhanced recovery after surgery/Conventional perioperative care interventions**																									
		**1**	**2**	**3**	**4**	**5**	**6**	**7**	**8**	**9**	**10**	**11**	**12**	**13**	**14**	**15**	**16**	**17**	**18**	**19**	**20**	**21**	**22**	**23**	**24**	**25**	**26**
Balzano et al. (23)	ERAS	√								√	√	√	√		√					√						√	√
	CPC									√	√	√	√		√												
French et al. (25)	ERAS																										
	CPC																										
Abu Hilal et al. (29)	ERAS				√						√				√		√			√						√	√
	CPC				√						√	√			√		√			√							
Nikfarjam et al. (33)	ERAS	√			√			√		√	√		√		√			√	√	√					√	√	√
	CPC							√		√	√															√	√
Braga et al. (31)	ERAS	√			√	√	√	√	√	√	√	√			√		√		√	√						√	√
	CPC	√			√	√		√		√	√	√					√		√	√						√	√
Coolsen et al. (7)	ERAS	√			√				√										√								
	CPC																										
Kobayashi et al. (32)	ERAS	√			√		√	√			√	√			√				√							√	√
	CPC							√			√	√			√												
Pillai et al. (8)	ERAS	√									√	√	√		√			√	√							√	√
	CPC										√		√					√								√	
Williamsson et al. (38)	ERAS	√			√					√	√		√		√					√	√				√	√	√
	CPC	√						√			√		√		√												
Richardson et al. (36)	ERAS							√											√		√		√			√	√
	CPC				√																					√	
Shao et al. (27)	ERAS											√														√	
	CPC											√															
Zouros et al. (39)	ERAS	√					√	√	√	√	√	√	√				√			√			√		√	√	√
	CPC																										
Shah et al. (37)	ERAS	√								√	√		√										√			√	√
	CPC																										
Partelli et al. (34)	ERAS	√					√		√	√	√	√					√	√		√		√				√	√
	CPC								√	√	√		√				√	√									√
Bai et al. (30)	ERAS	√					√			√	√		√		√								√				
	CPC	√									√		√		√												
Dai et al. (24)	ERAS	√					√				√	√					√			√			√			√	√
	CPC	√									√	√					√						√				
van der Kolk et al. (28)	ERAS	√			√					√	√	√					√	√		√							√
	CPC	√			√					√	√	√					√	√									√
Pecorelli et al. (35)	ERAS	√					√		√	√	√	√			√		√	√		√			√			√	√
	CPC	√								√	√	√					√	√									√
Kagedan et al. (26)	ERAS	√			√							√						√	√	√						√	√
	CPC																										

*ERAS, Enhanced Recovery after Surgery; CPC, conventional perioperative care; Items of Enhanced Recovery After Surgery/Fast-Track Surgery Interventions: 1 = Preoperative counseling, 2 = Perioperative biliary drainage, 3 = Preoperative smoking and alcohol consumption, 4 = Preoperative nutrition, 5 = Perioperative oral immunonutrition (IN), 6 = Oral bowel preparation, 7 = Preoperative fasting and preoperative treatment with carbohydrates, 8 = Preanaesthetic medication, 9 = Anti-thrombotic prophylaxis, 10 = Antimicrobial prophylaxis and skin preparation, 11 = Epidural analgesia, 12 = Intravenous analgesia Some evidence, 13 = Wound catheters and transversus abdominis plane block, 14 = Postoperative nausea and vomiting (PONV), 15 = Incision, 16 = Avoiding hypothermia, 17 = Postoperative glycaemic control, 18 = Nasogastric intubation, 19 = Fluid balance, 20 = Perianastomotic drain, 21 = Somatostatin analogs, 22 = Urinary drainage, 23 = Delayed gastric emptying, 24 = Stimulation of bowel movement, 25 = Postoperative artificial nutrition, 26 = Early and scheduled mobilization*.

### Quality Assessment of Included Studies

Cohort and case-control studies were both evaluated for bias based on the New-castle-Ottawa Scale (Supplemental Tables 2, 3). Among cohort studies, six studies received more than six stars, while the remaining study (25) received six stars. Among case-control studies, most articles obtained at least six stars, and only two articles received fewer than six stars. Therefore, most of the studies considered for this meta-analysis were of high quality.

### Primary Outcome Measures

#### Fistula

**Figure 2:**
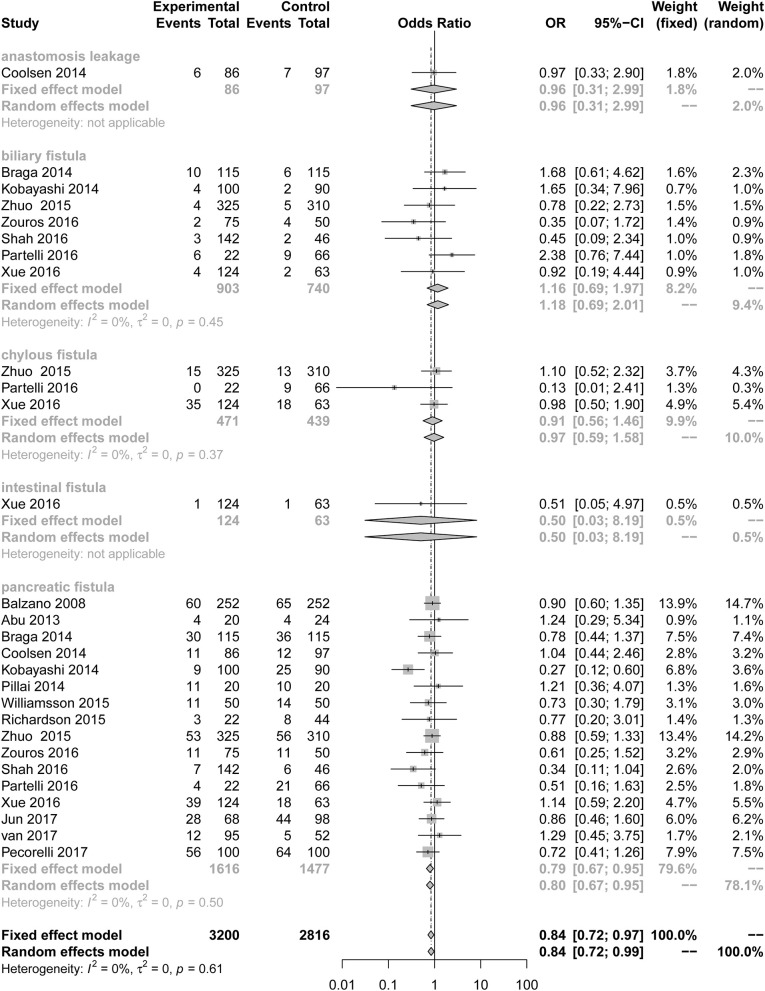
Forest plots demonstrating fistula of studies in terms of ERAS vs. CPC after pancreaticoduodenectomy by subgroup analysis.

Our results illustrate the incidence of complications comparing a multimodal ERAS protocol to conventional care. ERAS is associated with a decreased incidence of PF [Figure 2; number of comparisons reporting outcome (*n* = 16; OR = 0.79; 95% CI: 0.67–0.95; *P* for heterogeneity = 0.50, *I*^2^ = 0%)]. However, subgroup analysis of studies for other fistulas showed that the ERAS group did not differ significantly from the control group in the incidence of anastomosis leakage (*n* = 1; OR = 0.96; 95% CI: 0.31–2.99; heterogeneity is not applicable), biliary fistula (*n* = 7; OR = 1.16; 95% CI: 0.69 to 1.97; *P* for heterogeneity = 0.45, *I*^2^ = 0%), chylous fistula (*n* = 3; OR = 0.91; 95% CI: 0.56 to 1.46; *P* for heterogeneity = 0.37, *I*^2^ = 0%) and intestinal fistula (*n* = 1; OR = 0.50; 95% CI: 0.03 to 8.19; heterogeneity is not applicable). Sensitive analysis of the quality of the article was performed after removing two articles with less than six stars, and the conclusion is the same as before (*n* = 14; OR = 0.84; 95% CI: 0.72 to 0.98; *P* for heterogeneity = 0.51, *I*^2^ = 0%).

#### Infection

**Figure 3:**
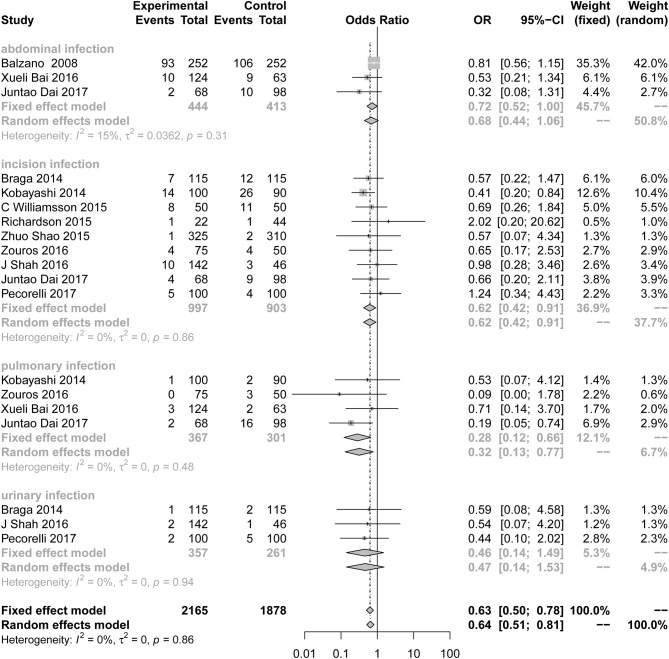
Forest plots demonstrating infection of studies in terms of ERAS vs. CPC after pancreaticoduodenectomy by subgroup analysis.

Compared to the control group, the incidence of infection (Figure 3; OR = 0.63; 95% CI: 0.50 to 0.78) was lower in the ERAS group. Different types of infections were mentioned in the studies, and the data for each infection are different. ERAS was associated with a lower incidence of incision infection (*n* = 9; OR = 0.62; 95% CI: 0.42 to 0.91) and pulmonary infection (*n* = 4; OR = 0.28; 95% CI: 0.12 to 0.66), but there were no significant differences in abdominal infection (*n* = 3; OR = 0.72; 95% CI: 0.52 to 1.00) and urinary infection (*n* = 3; OR = 0.46; 95% CI: 0.14 to 1.49) between the experimental group and the control group. No heterogeneity was found in this subgroup analysis (*I*^2^ = 0%, *P* = 0.86).

Sensitive analysis of the quality of the article was performed after removing two articles with less than six stars, and the conclusion is the same as before (*n* = 14; OR = 0.84; 95% CI: 0.72 to 0.98; *P* for heterogeneity = 0.51, *I*^2^ = 0%).

#### Delayed Gastric Emptying

**Figure 4:**
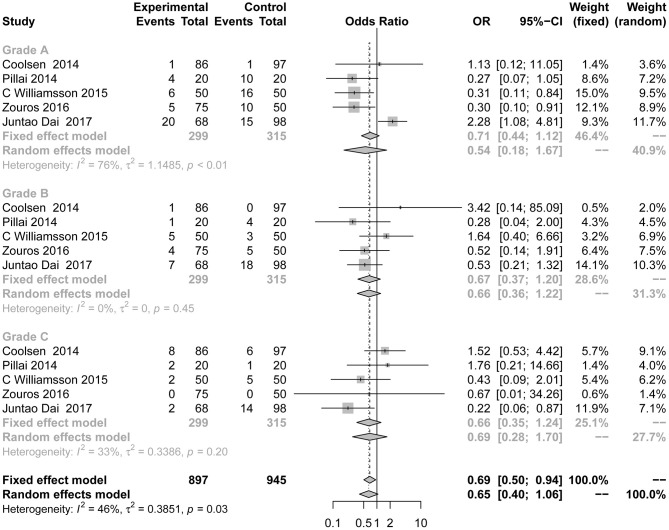
Forest plots demonstrating DGE of studies in terms of ERAS vs. CPC after pancreaticoduodenectomy by subgroup analysis.

Differences in the rates of DGE (Figure 4) were not consistently reduced in the ERAS group. There was also no significant difference between the control group and the experimental group in different grades of DGE. Five studies (7, 8, 24, 38, 39) reported DGE grade A (OR = 0.54; 95% CI: 0.18 to 1.67; *I*^2^ = 76%, *p* < 0.01), grade B (OR = 0.67; 95% CI: 0.37 to 1.20; *I*^2^ = 0%, *p* = 0.45), and grade C (OR = 0.66; 95% CI: 0.35 to 1.24; *I*^2^ = 33%, *p* = 0.20). There was moderate heterogeneity in this subgroup analysis (*I*^2^ = 46%, *p* = 0.03) using the random effects model.

#### Mortality

**Figure 5:**
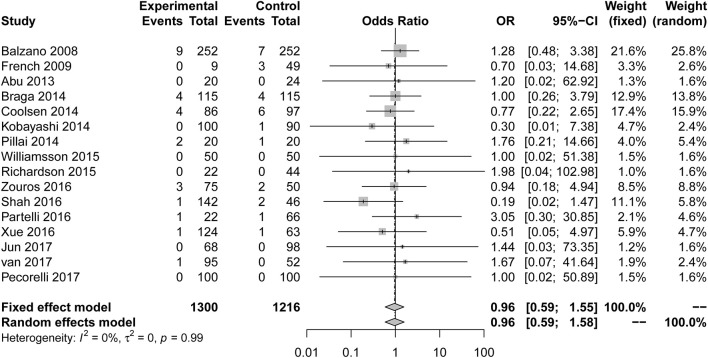
Forest plots demonstrating the mortality of studies in terms of ERAS vs. CPC after pancreaticoduodenectomy.

Sixteen studies (7, 8, 23–25, 28–32, 34–39) reported mortality as the primary outcome (Figure 5). The OR for mortality was 0.96 (95% CI: 0.59 to 1.55). Compared with the control group, the risk of mortality in the ERAS group was not significantly different. The heterogeneity determination of these studies using the fixed effect model was *I*^2^ = 0%, *P* = 0.99; therefore, no heterogeneity was found. After eliminating two articles with less than six stars in their quality scores, the result is as follows: OR = 0.94; 95% CI: 0.58 to 1.55; *P* for heterogeneity = 0.97, *I*^2^ = 0%.

#### Readmission

**Figure 6:**
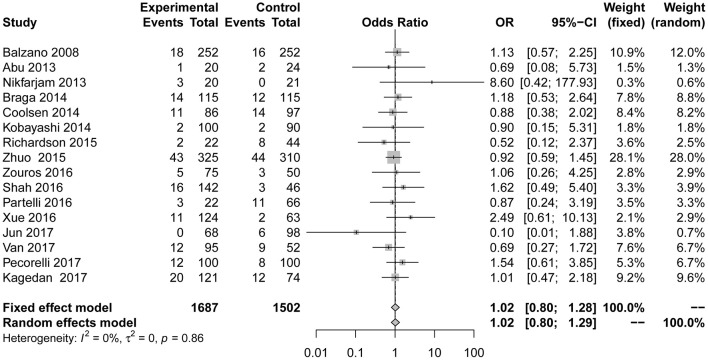
Forest plots demonstrating readmission of studies in terms of ERAS vs. CPC after pancreaticoduodenectomy by subgroup analysis.

The primary outcome measure readmission (Figure 6) was also used in 16 studies (7, 23, 24, 26–37, 39). No significant difference from the control group was found when evaluating the combination of all included studies (OR = 1.02; 95% CI: 0.80 to 1.28). No heterogeneity (*I*^2^ = 0%, P for heterogeneity = 0.86) using the fixed effect model was detected. After eliminating two articles with less than six stars in their quality scores, the result is as follows: OR = 1.03, 95% CI: 0.82 to 1.31; *P* for heterogeneity = 0.85, *I*^2^ = 0%.

#### Reoperation

**Figure 7:**
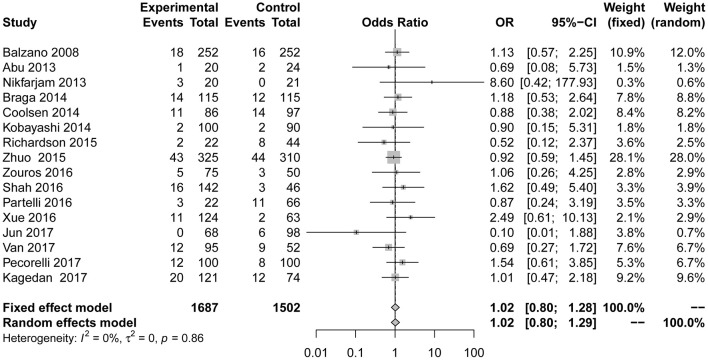
Forest plots demonstrating reoperation of studies in terms of ERAS vs. CPC after pancreaticoduodenectomy by subgroup analysis.

Reoperation data were shown in 8 studies (7, 23, 24, 28–31, 39). We found no evidence that reoperation (Figure 7) performed significantly differently between the two groups in the fixed effect model (OR = 0.82; 95% CI: 0.55 to 1.21). No heterogeneity (*I*^2^ = 0; *p* = 0.80) was detected.

#### Secondary Outcome Measures

**Figure 8:**
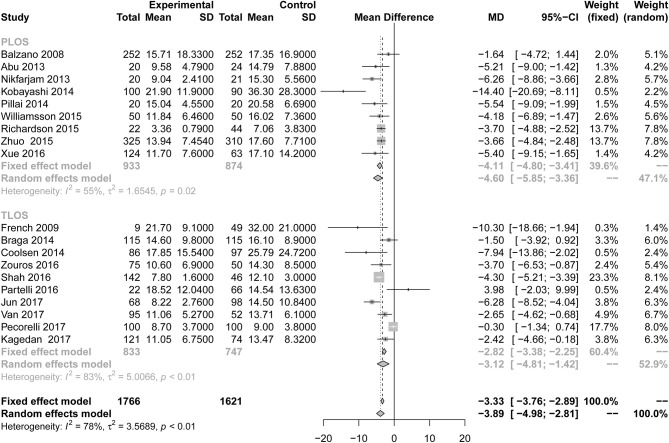
Forest plots demonstrating LOS of studies in terms of ERAS vs. CPC after pancreaticoduodenectomy by subgroup analysis.

**Figure 9:**
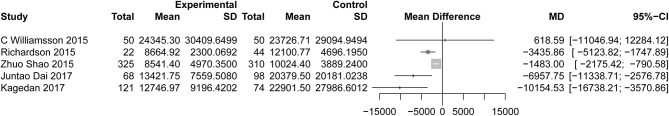
Forest plots demonstrating cost of studies in terms of ERAS vs. CPC after pancreaticoduodenectomy.

All studies reported the secondary outcome: LOS (MD = −3.89; 95% CI: −4.98 to −2.81; *I*^2^ = 78%, *p* < 0.01; Figure 8). Meta-analysis including 1,087 patients showed that patients in the ERAS group had a shorter postoperative LOS than those in the conventional group (MD = −4.60 days; 95% CI: −5.85 to −3.36), although a moderate degree of heterogeneity was observed (*I*^2^ = 55%, *P* = 0.02). Ten studies (7, 24–26, 28, 31, 34, 35, 37, 39) provided the data total LOS. The estimated mean for the meta-analysis of these studies was −3.12 days (95% CI: −4.81 to −1.42), indicating a significant reduction in the mean of total LOS for the ERAS patients compared with the conventional group. The statistical results of *I*^2^ (83%) showed highly heterogeneous research results in forest plots. Hospitalization costs (Figure 9) were reported by five studies and statistical analysis showed that ERAS protocols significantly reduced costs. Only one of the articles showed a lower cost in the control group. Pancreatic surgery can cost up to tens of thousands of dollars and costs at least several thousand dollars.

## Discussion

Progress in surgical techniques, improvements in equipment, technology, anesthesia, and perioperative care have contributed significantly to reducing the mortality after pancreatoduodenectomy; in most high-volume centers, the mortality rate is <5% (9). While reducing mortality, the emphasis now is on strengthening rehabilitation and reducing complications (8). Complications are a major reason for longer LOS. Previous studies have shown that reducing complications can reduce LOS. Some controversies regarding decreasing complications such as pancreatic fistula, infection and DGE using ERAS protocols after PD still persists.

A large number of data in this meta-analysis showed that ERAS and conventional groups did not significantly differ in the rates of mortality, reoperation, and readmission indicating that earlier discharge after implementation of the ERAS protocol did not affect patient morbidity (24). Most of the readmissions were due to complications, and slightly longer hospital stays can be greatly reduced (37). The results of this study suggest that the number of complications, such as PF and infection, can be safely decreased using ERAS protocols, especially with regard to incision and pulmonary infections. Reducing blood loss during surgery can reduce postoperative complications, especially suppurative infections (40). Because of the electronic laparoscopy used in some surgeries, the incision is smaller, the amount of bleeding is correspondingly reduced, and the chance of incision infection is greatly reduced. The reduction of pulmonary infection may be caused by early mobilization (41) and early removal of nasogastric tubes (42). In most surgeries, the nasogastric tube was removed 1 day after placement to monitor hemorrhage in all types of anastomosis. Prolonged placement of the nasogastric tube can lead to fever, pneumonia and atelectasis (37). The reduction in these complications is desirable because they are the most common complications in patients undergoing PD and constitute the dominant reasons for prolonged LOS and high hospital costs (43). Other types of fistula after operation have been investigated in this meta-analysis, such as anastomotic fistula, biliary fistula, chylous fistula and intestinal fistula. Perhaps owing to the small sample size, no statistical significance could be found. One study suggested early post-operative feeding may improve gastric emptying and peristalsis in the intestine, thereby reducing DGE (44). A subgroup analysis of DGE showed no significant correlation with DGE grade, independent of utilization of the ERAS program. This finding indicated that heterogeneity of DGE was mainly derived from grade A, but such a result did not indicate a limitation of ERAS.

Regarding secondary outcome measures, ERAS programs are associated with shorter LOS, both in the postoperative LOS and total LOS. From a patient perspective, the reduction in postoperative LOS is associated with reduced DGE rates and an earlier return to normal nutrition and enteric function, as well as lower levels of pain and a quicker return to preoperative levels of mobility, resulting in an overall improvement in the postoperative experience. One of the determining factors is the healthcare system depending on different cultural and economic environments. The variable may contribute to the higher heterogeneity observed in our analysis, which was different when analyzing only studies from western centers or Asian countries (13). Some of the reduced LOS is not just improvement of the hospital medical equipment, but includes the patients without the complications (39). The use of laparoscopic technique can make time shorter during operations (27). This result is consistent with a meta-analysis of pancreaticoduodenectomy showing a reduction in the LOS with 4 days (13). Hospitalization costs were lower in the experimental group than in the control group, independent of the country in which the treatment was received. Fewer complications and LOS correspondingly lead to fewer costs. Sometimes it is undeniable that doctors don't have a uniform level of expertise, and less experienced doctors need more tests to help diagnosis and patients spend more. One of the articles found that the most important economic effect associated with ERAS was the cost reduction in laboratory investigations, medical imaging, pharmaceuticals and patient food (26). There is no denying that laparoscopic surgery, or the use of robotic surgery, can have varying degrees of impact on the cost and recovery time of surgery. In this study, there was only one case of laparoscopic surgery and no robotic surgery.

Compared with the meta-analyses published in 2016 (13) and 2018 (14), we found consistency in LOS, rates of readmission, reoperation, and mortality. However, PF rates were lower for the ERAS group in our study. Additionally, incision infection and pulmonary infection rates were reduced in the ERAS group. DGE rates did not differ between the two groups in our study. According to the guideline for pylorus-preserving PDs, it has been shown that constructing the duodenojejunostomy in an antecolic (as opposed to a retro-colic) fashion results in reduced DGE (6). Thus, we need more data to certify that ERAS can decrease the rate of DGE. It should be noted that early postoperative oral intake does not worsen anastomotic leakage in colorectal surgery (45). Early postoperative oral intake has been avoided in patients undergoing PD with the concern that it may stimulate pancreatic exocrine secretion, resulting in an increased incidence of PF (32).

The purpose of ERAS protocols is to reduce patient stress; so it is important that guidelines mention several major measures: preoperative counseling with various information, avoiding oral bowel preparation and limiting fluid intake. The first measure can eliminate patients' preoperative anxiety (46), and the next one can decrease the incidence of anastomotic insufficiency (47), and liquid management can also reduce anastomotic fistula; this recommendation is also mentioned in the ERAS published in 2018. The included studies did not report the choice of incision at the surgeon's discretion, which should be of a length sufficient to ensure good exposure, so it cannot provide the evidence for clinical treatment. Pre-emptive use of nasogastric tubes postoperatively does not improve outcomes, and their use is not warranted routinely in the guidelines. An important measure is the early removal of the nasogastric duct, which can reduce the incidence of PF, consistently with the outcomes of many studies. Studies have shown that the carbohydrate beverage given to patients on the night before surgery and 2~4 h before surgery can alleviate the above stress response to some extent. To sum up, the ERAS program appears to be feasible in pancreaticoduodenectomy.

This meta-analysis not only provides evidence for using ERAS guidelines but also shows a new result regarding infection. ERAS can reduce incisions and lung infections. At the same time, the main outcome of this study was not LOS but the effect of the surgery itself, which has significant impact on clinical outcomes. The study incorporated all observational studies that contained large data groups to support the results reported and to increase the accuracy of the results.

This study has three main limitations: (1) it is unlikely that truly blinded, case-control studies regarding ERAS protocols will be performed due to a lack of feasibility. (2) It is very difficult to compare the incidence rates between different treatment centers according to the confirmed case, as the study reported the complication classification scheme (Clavien classification), and a suggestion for grading the complications based on the treatment intervention was to use a compound endpoint, which would reduce the required sample size study and improve objectivity and comparability. (3) Only two studies were randomized controlled trials (48, 49); therefore, data contained in these studies cannot be effectively analyzed.

## Conclusion

In conclusion, this meta-analysis showed a decrease in the rates of PF, infection, LOS and hospital costs without increasing the incidence of mortality, readmission, or reoperation in patients undergoing pancreatic duodenal surgery when ERAS protocols were applied in the patients' perioperative care. This is the time to promote the use of ERAS pathways as a protocol to restore patients' health after a complex and delicate surgery. With continued improvement in outcome results, ERAS protocols will attain the standard for primary abdominal surgeries.

## Data Availability

The datasets for this manuscript are not publicly available because all the data is in the manuscript. Requests to access the datasets should be directed to YF, fuyan_taihe0601@163.com.

## Author Contributions

CZ and YF had full access to all of the data in the study and took responsibility for the integrity of the data and the accuracy of the data analysis. JL, YC, and CZ designed the study. JL, YC, and Z-DH developed and tested the data collection forms. H-YG, Y-PW, and QZ acquired the data. YC, H-YG, and Z-DH conducted the analysis and interpreted the data. YC drafted the manuscript. CZ and YF had guarantor. All authors critically revised the manuscript.

### Conflict of Interest Statement

The authors declare that the research was conducted in the absence of any commercial or financial relationships that could be construed as a potential conflict of interest.
