# Supplementary material for: Impact of Enhanced Recovery After Surgery on Postoperative Recovery for Pancreaticoduodenectomy: Pooled Analysis of Observational Study
Source: Front Oncol. 2019 Jul 30;9:687. doi: 10.3389/fonc.2019.00687 (PMC6683725; doi:10.3389/fonc.2019.00687)
Supplement: Supplementary file 1 [file Data_Sheet_1.docx]

**Leagues of Supplemental Materials**

**Supplemental Method 1** Search strategy

**Supplemental Table 1** Reasons for excluding literatures

**Supplemental Table 2.** Assement of bias in case-control studies

**Supplemental Table 3.** Assement of bias in cohort studies

**Supplemental Method 1** Search strategy

**1. Ovid MEDLINE(R) Epub Ahead of Print, In-Process & Other Non-Indexed Citations, Ovid MEDLINE(R) Daily, Ovid MEDLINE and Versions(R)** 1946 to May 2 2018

(Enhanced Recovery After Surgery).mp. /(984)

(Enhanced Recovery Pathways).mp. /(119)

ERAS.mp. /(2018)

(Fast-track surgery).mp. /(376)

(FTS).mp. /(1160)

(Clinical pathway).mp. /(1646)

(Critical pathway).mp. /(730)

(Multimodal perioperative care).mp. /(26)

(Accelerated rehabilitation).mp. /(260)

1 or 2 or 3 or 4 or 5 or 6 or 7 or 8 or 9 /(7422)

(Pancreatectomy).mp. /(15060)

(Pancreaticoduodenectomy).mp. /(8924)

(Pancreatoduodenectomy).mp. /(2666)

(Pancreatoduodenal Resection).mp. /(148)

(Pancreatitis surgery).mp. /(241)

(Pancreatic Neoplasms).mp. /(65523)

(Pancreatic Cancer).mp. /(31508)

(Duodenectomy).mp. /(616)

(Whipple).mp. /(3693)

11 or 12 or 13 or 14 or 15 or 16 or 17 or 18 or 19 /(89521)

10 and 20 /(107)

**2. OVID Embase Classic+Embase** 1947 to May 01 2018

1. (Enhanced Recovery After Surgery).mp. /(1541)
2. (Enhanced Recovery Pathways).mp. /(189)
3. (ERAS).mp. /(4948)
4. (Fast-track surgery).mp. /(649)
5. (FTS).mp. /(1512)
6. (Clinical pathway).mp. /(8889)
7. (Critical pathway).mp. /(998)
8. (Multimodal perioperative care).mp. /(42)
9. (Accelerated rehabilitation).mp. /(356)
10. 1 or 2 or 3 or 4 or 5 or 6 or 7 or 8 or 9 /(17196)
11. (Pancreatectomy).mp. /(13891)
12. (Pancreaticoduodenectomy).mp. /(18775)
13. (Pancreatoduodenectomy).mp. /(4129)
14. (Pancreatoduodenal Resection).mp. /(198)
15. (Pancreatitis surgery).mp. /(89)
16. (Pancreatic Neoplasms).mp. /(4568)
17. (Pancreatic Cancer).mp. /(51702)
18. (Duodenectomy).mp. /(1092)
19. (Whipple).mp. /(3797)
20. 11 or 12 or 13 or 14 or 15 or 16 or 17 or 18 or 19 /(81715)
21. 10 and 20 /(197)

**3. CENTRAL, The Cochrane Library**, May 2018

Enhanced Recovery After Surgery /(637)

Enhanced Recovery Pathways /(125)

ERAS /(255)

Fast-track surgery /(490)

FTS /(57)

Clinical pathway /(6415)

Critical pathway /(966)

Multimodal perioperative care /(167)

Accelerated rehabilitation /(267)

#1 or #2 or #3 or #4 or #5 or #6 or #7 or #8 or #9 /(8021)

Pancreatectomy /(442)

Pancreaticoduodenectomy /(624)

Pancreatoduodenectomy /(150)

Pancreatoduodenal Resection /(4)

Pancreatitis surgery /(1230)

Pancreatic Neoplasms /(1593)

Pancreatic Cancer /(3173)

Duodenectomy /(27)

Whipple /(204)

#11 or #12 or #13 or #14 or #15 or #16 or #17 or #18 or #19 /(5136)

#10 and #20 in Trials /(124)

**4. ISIWeb of Science, Indexes=SCI-EXPANDED, SSCI, A&HCI, CPCI-S, CPCI-SSH, BKCI-S, BKCI-SSH, ESCI, CCR-EXPANDED, IC** May 01 2018

#1 TS=((Enhanced Recovery After Surgery) OR (Enhanced Recovery Pathways) OR ERAS OR (fast-track surgery) OR FTS OR (accelerated rehabilitation)) /(106168)

#2 TS=((Pancreatectomy) OR (Pancreaticoduodenectomy) OR (Pancreatoduodenectomy) OR (Pancreatoduodenal Resection) OR (Pancreatitis surgery) OR (Pancreatic Neoplasms) OR (Pancreatic Cancer) OR (duodenectomy) OR (Whipple)) /(84549)

#3 #1 And #2 /(548)

**Supplemental Table 1** Reasons for excluding literatures

| **Author** | **Year** | **Title** | **Reason** |
| --- | --- | --- | --- |
| Hakamada | 2004 | Impact of a Clinical Pathway for Pancreaticoduodenectomy on Cost Quality Outcomes and Patient Satisfaction | The study design didn't meet the inclusion criteria. |
| Wichmann | 2006 | A prospective clinical feasibility study for multimodal "fast track" rehabilitation in elective pancreatic cancer surgery | The outcomes didn't meet the inclusion criteria. |
| Berberat | 2007 | Fast track-different implications in pancreatic surgery | The study design didn't meet the inclusion criteria. |
| Bruns | 2007 | Fast track pancreatic cancer surgery | The outcomes didn't meet the inclusion criteria. |
| Nakamura | 2009 | Economic impact of fast-track surgery after pancreaticoduodenectomy | The study design didn't meet the inclusion criteria. |
| Wersch Van | 2010 | Enhanced recovery after surgery programme for pancreatic surgery - An assessment of hospital stay, readmissions and complications | The study design didn't meet the inclusion criteria. |
| Alistar | 2012 | Clinical pathways for pancreatic neuroendocrine tumors | The outcomes didn't meet the inclusion criteria. |
| Robertson | 2012 | Implementation of an enhanced recovery programme following pancreaticoduodenectomy | The outcomes didn't meet the inclusion criteria. |
| Shchepotin | 2012 | Efficacy of multimodal program "fast track surgery" in the surgical treatment for pancreatic cancer patients | The study design didn't meet the inclusion criteria. |
| Jin | 2013 | Fast-track recovery after pancreatoduodenectomy: A prospective randomized controlled study | The study design didn't meet the inclusion criteria. |
| Yamaki | 2013 | The clinical role of critical pathway implementation for pancreaticoduodenectomy in 179 patients | The study design didn't meet the inclusion criteria. |
| Kovalenko | 2014 | Fast track recovery pathways after pancreatoduodenectomy: Initial clinical experience | The study design didn't meet the inclusion criteria. |
| Tinti | 2014 | Enhanced recovery after surgery in pancreatic surgery: Initial experience in a high volume center | The study design didn't meet the inclusion criteria. |
| Wei | 2014 | Development of an evidence-based clinical pathway for patients undergoing pancreaticoduodenectomy | The study design didn't meet the inclusion criteria. |
| Galli | 2015 | Enhanced Recovery After Surgery (ERAS (R)) multimodal programme as experienced by pancreatic surgery patients: Findings from an Italian qualitative study | The outcomes didn't meet the inclusion criteria. |
| Lyadov | 2015 | Enhanced recovery pathways in pancreatoduodenectomy: 2 year experience from a Russian high-volume center | The study design didn't meet the inclusion criteria. |
| Manatakis | 2015 | Implementation of an enhanced recovery protocol in pancreatic surgery-preliminary results | The study design didn't meet the inclusion criteria. |
| Morales Soriano | 2015 | Outcomes of an enhanced recovery after surgery programme for pancreaticoduodenectomy | The study design didn't meet the inclusion criteria. |
| Nct | 2015 | Accelerated Recovery Pathway for Discharge After Surgery in Patients With Pancreatic Cancer | The outcomes didn't meet the inclusion criteria. |
| Nct | 2015 | The Effect of ERAS on Pancreaticoduodenectomy | The study design didn't meet the inclusion criteria. |
| Park | 2015 | Eras after pancreatoduodenectomy | The study design didn't meet the inclusion criteria. |
| Rashid | 2015 | Outcomes of a clinical pathway for borderline resectable pancreatic cancer | The outcomes didn't meet the inclusion criteria. |
| Saunders | 2015 | Predicting delayed gastric emptying after pancreaticoduodenectomy for enhanced recovery after surgery | The study design didn't meet the inclusion criteria. |
| Sutcliffe | 2015 | Implementation of an Enhanced Recovery Pathway After Pancreaticoduodenectomy in Patients with Low Drain Fluid Amylase | The study design didn't meet the inclusion criteria. |
| Velu | 2015 | Enhanced recovery after surgery protocols as a management strategy following major pancreatic surgery | The study design didn't meet the inclusion criteria. |
| Bond-Smith | 2016 | Enhanced recovery protocols for major upper gastrointestinal, liver and pancreatic surgery | The study design didn't meet the inclusion criteria. |
| Joliat | 2016 | Implementation of an enhanced recovery program for pancreas head resection is highly costeffective-results of a costbenefit analysis of 161 patients | The study design didn't meet the inclusion criteria. |
| Kazama | 2016 | Safety and feasibility of enhanced recovery after surgery in the patients underwent pancreaticoduodenectomy for hepatobiliary and pancreatic malignancy | The study design didn't meet the inclusion criteria. |
| Kovalenko | 2016 | Implication of fast track protocol in pancreatoduodenectomy | The study design didn't meet the inclusion criteria. |
| Miao Y. | 2016 | [The possibility and feasibility of implementation of enhanced recovery after surgery program in pancreatic surgery] | The study design didn't meet the inclusion criteria. |
| Morgan | 2016 | Enhanced Recovery after Surgery Protocols Are Valuable in Pancreas Surgery Patients | The outcomes didn't meet the inclusion criteria. |
| Palani Velu | 2016 | Enhanced recovery protocols after pancreaticoduodenectomy improve outcome but not through modulation of the systemic inflammatory response | The outcomes didn't meet the inclusion criteria. |
| Rashid | 2016 | Outcomes of a Clinical Pathway for Borderline Resectable Pancreatic Cancer | The outcomes didn't meet the inclusion criteria. |
| Deng, Xiaxing | 2017 | Modified protocol for enhanced recovery after surgery is beneficial for Chinese cancer patients undergoing pancreaticoduodenectomy | The study design didn't meet the inclusion criteria. |
| Marquez Mesa | 2017 | Nutrition management in enhanced recovery after abdominal pancreatic surgery | The outcomes didn't meet the inclusion criteria. |
| Roberts | 2017 | A reduced time to surgery within a 'fast track' pathway for periampullary malignancy is associated with an increased rate of pancreatoduodenectomy | The population didn't meet the inclusion criteria. |
| Su, Wei | 2017 | A hospital-to-home evaluation of an enhanced recovery protocol for elective pancreaticoduodenectomy in China: A cohort study | The study design didn't meet the inclusion criteria. |
| Tremblay St-Germain | 2017 | The impact of a clinical pathway on patient postoperative recovery following pancreaticoduodenectomy | The study design didn't meet the inclusion criteria. |
| Agarwal | 2018 | Improved Outcomes in 394 Pancreatic Cancer Resections: the Impact of Enhanced Recovery Pathway | The study design didn't meet the inclusion criteria. |
| Sheel | 2018 | Enhanced Recovery after Surgery (ERAS) reduces the incidence of Delayed Gastric Emptying (DGE) following Pancreaticoduodenectomy | The study design didn't meet the inclusion criteria. |

**Supplemental Table 2** Assement of bias in case-control studies

| **Authors** | **Selection** | | | |  | **Comparability** |  | **Exposure** | | |  | **Total** |
| --- | --- | --- | --- | --- | --- | --- | --- | --- | --- | --- | --- | --- |
|  | **Is the Case Definition Adequate?** | **Representativeness of the Cases** | **Selection of Controls** | **Definition of Controls** |  | **Comparability of Cases and Controls on the Basis of the Design or Analysis** |  | **The same method was used to determine the exposure factors in both cases and controls** | **Ascertainment of Exposure** | **Non-Response Rate** |  |  |
| Abu Hilal 2013 | ★ | ★ |  | ★ |  | ★ |  | ★ | ★ |  |  | 6 |
| Nikfarjam 2013 | ★ | ★ |  | ★ |  | ★ |  | ★ | ★ |  |  | 6 |
| Braga 2014 | ★ | ★ |  | ★ |  | ★★ |  | ★ | ★ |  |  | 7 |
| Coolsen 2014 | ★ | ★ |  | ★ |  | ★ |  | ★ | ★ |  |  | 6 |
| Kobayashi 2014 | ★ | ★ |  | ★ |  | ★ |  | ★ | ★ |  |  | 6 |
| Williamsson  2015 | ★ | ★ |  | ★ |  | ★ |  |  |  |  |  | 4 |
| Richardson 2015 | ★ |  |  | ★ |  | ★ |  | ★ | ★ |  |  | 5 |
| Zouros 2016 | ★ | ★ |  | ★ |  | ★ |  | ★ | ★ |  |  | 6 |
| J Shah 2016 | ★ | ★ |  | ★ |  | ★ |  | ★ | ★ |  |  | 6 |
| Partelli 2016 | ★ | ★ |  | ★ |  | ★ |  | ★ | ★ |  |  | 6 |
| Xueli Bai 2016 | ★ | ★ |  | ★ |  | ★ |  | ★ | ★ |  |  | 6 |
| Pecorelli 2017 | ★ | ★ |  | ★ |  | ★ |  | ★ | ★ |  |  | 6 |

**Note:** The Newcastle-Ottawa Scale (NOS) of Case-control studies (A maximum of two stars can be given for Comparability and one star for each numbered item within the Selection and Exposure categories).

**Supplemental Table 3** Assement of bias in cohort studies

| **Authors** | **Selection** | | | |  | **Comparability** |  | **Exposure** | | |  | **Total** |
| --- | --- | --- | --- | --- | --- | --- | --- | --- | --- | --- | --- | --- |
|  | **Representativeness of the exposed cohort** | **Selection of the nonexposed cohort** | **Ascertainment of exposure** | **Demonstration that outcome of interest was not present at start of study** |  | **Comparability of Cases and Controls on the Basis of the Design or Analysis** |  | **Assessment of outcome** | **Was follow-up long enough for outcomes to occur** | **Adequacy of follow up of cohorts** |  |  |
| Balzano 2008 | ★ |  | ★ | ★ |  | ★ |  | ★ | ★ | ★ |  | 7 |
| French 2009 | ★ |  | ★ | ★ |  |  |  | ★ | ★ | ★ |  | 6 |
| Pillai 2014 | ★ |  | ★ | ★ |  | ★ |  | ★ | ★ | ★ |  | 7 |
| Zhuo Shao 2015 | ★ | ★ | ★ | ★ |  | ★ |  | ★ |  | ★ |  | 7 |
| Juntao Dai 2017 | ★ | ★ | ★ | ★ |  | ★ |  | ★ | ★ | ★ |  | 8 |
| Kolk 2017 | ★ | ★ | ★ | ★ |  | ★ |  | ★ | ★ | ★ |  | 8 |
| Kagedan 2017 | ★ | ★ | ★ | ★ |  | ★ |  | ★ | ★ | ★ |  | 8 |

**Note:** The Newcastle-Ottawa Scale (NOS) of Cohort studies (A maximum of two stars can be given for Comparability and one star for each numbered item within the Selection and Exposure categories).
